# Sensitive quantification of *Clostridium perfringens* in human feces by quantitative real-time PCR targeting alpha-toxin and enterotoxin genes

**DOI:** 10.1186/s12866-015-0561-y

**Published:** 2015-10-19

**Authors:** Ravinder Nagpal, Kiyohito Ogata, Hirokazu Tsuji, Kazunori Matsuda, Takuya Takahashi, Koji Nomoto, Yoshio Suzuki, Kazunari Kawashima, Satoru Nagata, Yuichiro Yamashiro

**Affiliations:** Probiotics Research Laboratory, Juntendo University Graduate School of Medicine, Tokyo, Japan; Yakult Central Institute, Tokyo, Japan; Yakult Honsha European Research Center for Microbiology, Ghent-Zwijnaarde, Belgium; Department of Sports Science, Juntendo University School of Health and Sports Sciences, Chiba, Japan; Gonohashi Obstetrics and Gynecology Hospital, Tokyo, Japan; Department of Pediatrics, School of Medicine, Tokyo Women’s Medical University, Tokyo, Japan

**Keywords:** Alpha-toxin, *Clostridium perfringens*, Enterotoxin, Intestinal microbiota, Gut pathogens, Phospholipase C, Quantitative PCR

## Abstract

**Background:**

*Clostridium perfringens* is a widespread pathogen, but the precise quantification of this subdominant gut microbe remains difficult due to its low fecal count (particularly in asymptomatic subjects) and also due to the presence of abundant polymerase-inhibitory substances in human feces. Also, information on the intestinal carriage of toxigenic *C. perfringens* strains in healthy subjects is sparse. Therefore, we developed a sensitive quantitative real-time PCR assays for quantification of *C. perfringens* in human feces by targeting its α-toxin and enterotoxin genes. To validate the assays, we finally observed the occurrence of α-toxigenic and enterotoxigenic *C. perfringens* in the fecal microbiota of healthy Japanese infants and young adults.

**Methods:**

The *plc*-specific qPCR assay was newly validated, while primers for 16S rRNA and cpe genes were retrieved from literature. The assays were validated for specificity and sensitivity in pre-inoculated fecal samples, and were finally applied to quantify *C. perfringens* in stool samples from apparently healthy infants (n 124) and young adults (n 221).

**Results:**

The qPCR assays were highly specific and sensitive, with a minimum detection limit of 10^3^ bacterial cells/g feces. Alpha-toxigenic *C. perfringens* was detected in 36 % infants and 33 % adults, with counts ranging widely (10^3^-10^7^ bacterial cells/g). Intriguingly, the mean count of α-toxigenic *C. perfringens* was significantly higher in infants (6.0 ± 1.5 log_10_ bacterial cells/g), as compared to that in adults (4.8 ± 1.2). Moreover, the prevalence of enterotoxigenic *C. perfringens* was also found to be significantly higher in infants, as compared to that in adults. The mean enterotoxigenic *C. perfringens* count was 5.9 ± 1.9 and 4.8 ± 0.8 log_10_ bacterial cells/g in infants and adults, respectively.

**Conclusions:**

These data indicate that some healthy infants and young adults carry α-toxigenic and enterotoxigenic *C. perfringens* at significant levels, and may be predisposed to related diseases. Thus, high fecal carriage of toxigenic *C. perfringens* in healthy children warrants further investigation on its potential sources and clinical significance in these subjects. In summary, we present a novel qPCR assay for sensitive and accurate quantification of α-toxigenic and enterotoxigenic *C. perfringens* in human feces, which should facilitate prospective studies of the gut microbiota.

**Electronic supplementary material:**

The online version of this article (doi:10.1186/s12866-015-0561-y) contains supplementary material, which is available to authorized users.

## Background

*Clostridium perfringens* is one of the most widely dispersed opportunistic pathogens [1] and is well known to produce a number of toxins to cause several forms of histotoxic and enteric diseases in humans and animals [2]. Based on the production of four major toxins i.e., alpha, beta, epsilon and iota, it is categorized into five toxin-types viz. A, B, C, D and E [3]. While it is ambiguous why *C. perfringens* produces so many diverse toxins, it is well known that it uses chromosomally-encoded α-toxin (which has phospholipase C (*plc*) and sphingomyelinase activities with hemolytic, necrotic and lethal abilities) as a chief virulent factor and key mediator for most of *C. perfringens*-associated diseases [4]. Alpha-toxin is produced by all *C. perfringens* toxin-types but type A produces higher amounts than the other types [1]. In addition, few *C. perfringens* strains also harbor *cpe* (*C. perfringens* enterotoxin, a clinically important enterotoxin) gene which is responsible for most of the *C. perfringens* food-poisoning outbreaks and diarrheal cases [5]. Under favorable conditions, the organism can cause gas gangrene, food-poisoning and gastrointestinal illnesses including antibiotic-associated diarrhea, sporadic diarrhea, and nosocomial diarrheal diseases in humans [6–9]. However, in spite of their ubiquitous environmental distribution and implication in various food-poisoning outbreaks worldwide [10, 11], very little information is available on the relative occurrence of α-toxigenic and enterotoxigenic strains of *C. perfringens* in general populations, particularly in Japan where *C. perfringens* food poisoning has been ranked as third or fourth most common food poisoning [12–14].

Generally, a high *C. perfringens* count (>10^6^ cfu/g feces) is considered as an indicator of *C. perfringens* food-poisoning [15–18]. *C. perfringens* in fecal samples has traditionally been detected by conventional microbiological culture methods, biochemical analyses, tissue culture cytotoxicity assays, mouse bioassays or immunosorbent assays [19–21], but these methods are laborious and time-consuming. Culture of *C. perfringens* is not recommended for diagnosis, mainly because of relative ubiquity of the bacterium in human feces. Further, since not all isolates are toxigenic, specific toxigenic strains that coexist with a larger population of non-toxigenic strains may be overlooked. Biochemical tests are also incapable of distinguishing different types of *C. perfringens*. As a consequence, traditional clinical diagnosis of enteric pathogens by conventional methods lack the competence for large-scale investigations and the adequate precision and sensitivity required for low-level sub-clinical detection. Several immunosorbent assays have also been developed, of which some are also commercially available for detection of clostridial toxins including *C. perfringens* toxins [20, 22, 23]; however, these assays are not as sensitive as traditional methods and often produce false-negative results [24–26]. Further, these toxin-specific immunosorbent assays may overlook the samples of those subjects who are carrying toxigenic bacterium but in whom the toxin is not produced [22]. Furthermore, the toxin may not be detected, for instance, if it is a modified toxin or neutralized by host factors [24]. Therefore, to avoid such discrepancies, the potential of PCR-based methods with specific gene targets has been explored. Rapid and specific detection has been achieved by molecular approaches based on PCR, and it has been used as a reliable tool to detect *C. perfringens* within complex microbial backgrounds such as complex food products and fecal samples [27–32]. However, since complex samples such as human feces contain numerous polymerase-inhibitory substances that affect the sensitivity of PCR reactions [33, 34], majority of the available methods require pure cultures or pre-incubation steps in order to enumerate lower counts (< 10^5^ cfu/g) of toxigenic strains of *C. perfringens*, particularly in the feces of asymptomatic subjects.

In this context, we developed a rapid and sensitive qPCR assay to detect and quantify *C. perfringens* directly in human fecal samples by using a novel primer pair targeting the chromosomally-located *plc* (α-toxin) gene. The assay was coupled with a commercially available PCR buffer Ampdirect® Plus (Shimadzu, Japan) as a measure to allow more efficient PCR amplification by neutralizing the effect of inhibitory substances present in biological samples [35]. The method was validated for its specificity and sensitivity against various targeted (*C. perfringens*) and non-targeted (non-*C. perfringens* and non-clostridial species) bacterial strains, and also for detection efficiency and reproducibility in artificially inoculated fecal samples. The final step in the analytical validation of the assay was its application to detect and quantify *C. perfringens* in fecal samples from 345 apparently healthy Japanese subjects (infants, *n* 124; adults, *n* 221). In addition to *plc* gene, the samples were also analyzed for 16S rRNA and chromosomal *cpe* genes. Herein, we provide a specific, sensitive and rapid assay for quantitative enumeration of toxigenic *C. perfringens* in human feces.

## Methods

### Primer design

The *plc* gene was targeted for the design of primer. The sequence of the gene was procured from GenBank, National Center for Biotechnology Information (NCBI) (http://www.ncbi.nlm.nih.gov/genbank/). Multiple alignment of the gene sequences was performed with the CLUSTAL_X program (http://clustalx.software.informer.com) [36] by using *plc* gene sequences from more than 25 strains of *C. perfringens* including the three reference strains*,* ATCC 13124^T^ (GenBank accession number: NC_008261; Gene ID: 4201274), Str. 13 (GenBank accession number: NC_003366; Gene ID: 988262) and SM101 (GenBank accession number: NC_008262; Gene ID: 4205371). After comparison of the sequences *in silico*, target sites for *C. perfringens plc*-specific detection were identified and a putative oligonucleotide primer set was designed following the primer designing instructions from user guide catalog (http://www3.appliedbiosystems.com/cms/groups/mcb_support/documents/generaldocuments/cms_041053.pdf). The primer was subsequently checked for potential cross-reactivity with the BLAST (Basic Local Alignment Search Tool) database search application (http://www.ncbi.nlm.nih.gov/BLAST), and for preliminary sensitivity and specificity by qPCR with extracted DNA fractions from 30 *Clostridium* and non-clostridia strains (Table 1 and 2). For the analysis of *cpe* gene, the primer pair reported previously [37] was used to amplify a 154 bp product from the chromosomal *cpe* locus (Table 1). The details of 16S rRNA gene-specific primer pair have previously been reported [38] (Table 1).

### Bacterial strains and culture conditions

The bacterial strains used in the present investigation included 8 *C. perfringens* and 22 non-*C. perfringens* strains, as listed in Table 2. The strains were type strains either from the American Type Culture Collection (ATCC) or Japan Collection of Microorganisms (JCM). The strain *C. perfringens* C052-1 was previously isolated and characterized from human feces in the author’s laboratory at Yakult Central Institute. All the Clostridia strains were routinely grown in modified Gifu anaerobic broth (Nissui Pharmaceutical Co., Ltd., Tokyo, Japan) supplemented with 1 % glucose (Glu-mGAM) at 37 °C for 24 h under anaerobic conditions. All anaerobic manipulations were performed in an anaerobic glove box (Coy Laboratory Products Inc., Grass Lake, MI). Total bacterial cell counts were determined by DAPI staining, according to the method described previously [39].

### Primary treatment of fecal samples

Fresh fecal specimens were collected from five healthy adult male subjects for spiking tests, and from 124 infants and 221 young adults for analytical validation of the qPCR assays (details of the subjects are provided in subsequent sections). Immediately after defecation, a spoonful of feces (0.5 to 1.0 g) was collected into a fecal collection tube (Sarstedt AG & Co., Numbrecht, Germany), and the tubes were stored at −80 °C until use for the pretreatment for qPCR. Primary treatment of feces for total DNA extraction was done as follows. Each fecal sample was weighed, suspended in 9 volumes of sterile PBS (−) to make 10-fold (v/w) dilution, and was washed twice with PBS (−) by centrifugation at 12,000 × *g* at 4 °C for 5 min. About equal volume of glass beads (2.5 mm, diameter), as that of fecal sample, were added to the diluted samples, and the mixture was subjected to vigorous vortex to make a uniform fecal homogenate which was then divided into 200 μl aliquots and stored at −80 °C until DNA extraction.

### DNA extraction

DNA extraction was performed according to the method previously described [40], with minor modifications. Briefly, the thawed sample (200 μl aliquots of pure cultures or primary treated fecal samples) was mixed with 250 μl of extraction buffer (100 mM Tris–HCl, 40 mM EDTA; pH 9.0), 50 μl of 10 % sodium dodecyl sulfate, 300 mg of glass beads (diameter, 0.1 mm), and 500 μl of Tris-EDTA-saturated phenol, and the mixture was subjected to vigorous vortexing for 15 min using a ShakeMaster Auto BMS-A15 (Bio Medical Science Inc., Tokyo, Japan). After phenol–chloroform purification and isopropanol precipitation of the sample, the nucleic acid fraction was suspended in 0.1 ml of TE (pH 8.0).

### Real-time qPCR assay

The specific primer sets for 16S rRNA, *plc* and *cpe* gene (as described in Table 1) were used. For qPCR without Ampdirect® plus (Shimadzu, Japan), each reaction mixture (20 μl) was composed of 1 × PCR buffer (TaKaRa Bio Inc., Shiga, Japan), each dNTP at a concentration of 200 μM, MgCl_2_ solution at a concentration of 2.5 mM, a 1:75,000 dilution of SYBR green I (BioWhittaker Molecular Applications, Rockland, ME), Takara Taq^TM^ (TaKaRa Bio Inc., Shiga, Japan) at a concentration of 0.02 units/μl, TaqStart antibody (Clontech, Palo Alto, CA) at a concentration of 5.5 ng/μl, and 5 μl template DNA. Each primer set was added at a final concentration of 0.2 μM. For qPCR with Ampdirect® plus (Amp-qPCR), each reaction mixture (20 μl) was composed of 1 × Ampdirect® plus buffer, a 1:75,000 dilution of SYBR green I, Takara Taq^TM^ at a concentration of 0.02 units/μl, TaqStart antibody at a concentration of 5.5 ng/μl, and 5 μl template DNA. Each primer set was added at a final concentration of 0.2 μM. The amplification program consisted of one cycle at 95 °C for 5 min, followed by 50 cycles at 94 °C for 20 s, 55/60 °C for 20 s, and 72 °C for 50 s. The fluorescent products were detected at the last step of each cycle. For determination of specificity of the amplifications, a melting curve analysis was performed in concurrence with each PCR runs by slow heating at temperatures from 60 to 95 °C, with fluorescence obtained at 0.2 °C/s. qPCR amplification and detection were performed in 96-well optical plates (Cat. No. 3754; Corning Inc. Life Sciences, Japan) with an ABI PRISM 7500 sequence detection system (version: 1.4.0; Applied Biosystems, Foster City, CA). The plates were sealed with optical-quality adhesive films (Cat. No. 547-KTS-HC-P; Watson Bio-Lab Co., Ltd., Japan). The MIQE (Minimum Information for the publication of real-time Quantitative PCR Experiments) guidelines [41] were followed for the description of experimental designs and results.

### Determination of primer specificity

The specificity of the newly-designed *plc* primer set and the reported *cpe* primer set was tested on several target and non-target reference strains, as listed in Table 2. Total DNA fractions extracted from the bacterial cells of each strain at a dose corresponding to 10^5^ cells were assessed for qPCR by using each primer set. Using the standard curve from *C. perfringens* ATCC 13124^T^ or *C. perfringens* ATCC 12917 obtained as described above, the amplified signal was judged to be positive when it was more than that of 10^4^ standard cells and negative when it was less than that of 10^−1^ standard cells. The amplified signal was defined as negative when the corresponding melting curve had a peak different from that of the standard strain. The specificity of 16S rRNA gene-specific primer has previously been reported by using Reverse Transcription-qPCR [38].

### Determination of qPCR sensitivity

*C. perfringens* ATCC 13124^T^ or *C. perfringens* ATCC 12917 was cultivated in Glu-mGAM broth. Bacterial counts were determined microscopically by DAPI staining and DNA was extracted from culture samples in the early stationary phase (24 h). Serial DNA dilutions corresponding to bacterial (DAPI) counts ranging from 10^0^ to 10^5^ cells were assessed by Amp-qPCR assays, and the standard curves were established. The range of DNA concentrations at which there was linearity with the *C*_*q*_ value was confirmed (*R*^2^ > 0.99).

### Determination of bacterial counts by qPCR

For identification of the target bacterial population in the fecal samples, 1/20, 1/200, 1/2,000 portion of the DNA extracts and 1/2,000, 1/20,000, 1/200,000 portion of the DNA extracts were subjected to Amp-qPCR and qPCR, respectively, and the *C*_*q*_ values in the linear range of the assay (10^0^ to 10^5^ cells) were applied to the analytical curve generated in the same experiment to obtain the corresponding bacterial count in each nucleic acid sample, which was converted to the count per g of feces. Multiple NTCs were included with every assay. The *C*_*q*_ values and the corresponding copy numbers for standards were included in the standard curve construction when the *C*_*q*_ values of standard were smaller than those of NTCs by 2 or more, and the LOD was defined as the smallest bacterial number in each standard curve.

### Quantification of *C. perfringens* spiked in human feces by qPCR

Fresh fecal samples were collected from five healthy adult males (ages, 28, 37, 41, 42, and 48 years) whose samples had previously been verified by previously established 16S rRNA gene-specific assay to be Amp-qPCR-negative (< 10^3^ cells/g feces) for *C. perfringens* [38]. The samples were subjected to the primary treatment as described above. It may be noted that for spiking tests, the samples were first suspended in 4 volumes of sterilized PBS (−) to make a 5-fold (v/w) dilution and were then mixed with an equal amount of bacterial suspension to obtain a 10-fold (v/w) diluted fecal suspension. An overnight grown active culture of *C. perfringens* ATCC 13124^T^ or *C. perfringens* ATCC 12917 was counted by DAPI staining and serially diluted in sterilized PBS (−) to make a series of concentrations from 2 × 10^7^ to 2 × 10^2^ cells/ml, and 500 μl of these bacterial dilutions was spiked into corresponding 500 μl of fecal homogenates to make final concentrations ranging from 1 × 10^8^ to 1 × 10^3^ cells/g of feces. DNA fractions were extracted from 200 μl of each spiked sample and were assessed by qPCR and Amp-qPCR assays, as described above. The *C*_*q*_ values obtained by the three primers were applied to the standard curve generated with serial dilution series of *C. perfringens* ATCC 13124^T^ (16S rRNA gene and *plc*) or *C. perfringens* ATCC 12917 (*cpe*) DNA corresponding to 10^0^ to 10^5^ cells per reaction to determine the qPCR counts. A melting curve analysis was performed after amplification to distinguish the target from the non-targeted PCR products. For determination of the number of bacteria in fecal samples, three serial dilutions of each extracted DNA sample (5 μl) were used for qPCR, and the *C*_*q*_ values in the linear range of the assay were applied to the standard curve generated in the same experiment to obtain the corresponding number of bacteria in each nucleic acid sample and then converted to the number of bacteria per gram of feces.

### Detection of *C. perfringens* in clinical samples

The newly-developed *plc*-specific Amp-qPCR assay was used to quantify *C. perfringens* in 345 fecal samples from apparently healthy infants (*n* 124; M 70; F 54; age: 167 ± 3.3 days; age range: 161–178 days) and young adults (*n* 221; M 153, F 68; age: 18.8 ± 0.9 years; age range: 18–22 years). The infants’ samples were obtained from Gonohashi Obstetrics and Gynecology Hospital, Koto-ku, Tokyo; and the adults’ samples were from students enrolled at Juntendo University Graduate School of Sports Medicine, Tokyo. Informed written consent was obtained from all the subjects or their legal representatives. The study designs of infant and adult subjects were approved by the ethical committees of Yakult Central Institute and Juntendo University Graduate School of Medicine, respectively. Immediately after defecation, samples were collected as per the method described above. The infant samples were kept at 4 °C in a cooling box with refrigerants and sent immediately to Yakult Central Institute where these were stored at 4 °C until the primary treatment. The adults’ samples were stored at −80 °C at the site of collection, and were transported in a frozen state to the Yakult Central Institute where these were stored at −80 °C until use for the primary treatment. The samples were subjected to the primary treatment and nucleic acid extraction, as described above. DNA samples were finally subjected to the newly-developed Amp-qPCR assay by using *plc*-specific primer pair, and were also analyzed simultaneously by using a previously validated 16S rRNA gene-specific primer pair for comparison purposes and also by *cpe*-specific primer pair for the analysis of enterotoxigenic strains (Table 1) by Amp-qPCR.

### Statistical analyses

Results of log-transformed bacterial count (log_10_ cells/g feces) are expressed as mean ± standard deviation. The program R (http://www.r-project.org) was used for statistical analyses. Mann–Whitney *U* test and Fisher’s exact probability test were used to calculate the mean differences in the fecal counts and the detection rates (prevalence), respectively. *P* < 0.05 was considered statistically significant.

## Results

### Specificity of the primer sets

The specificity of *plc*- and *cpe*-targeted primers was tested *in vitro* by using DNA fractions extracted from several target and non-target bacterial strains corresponding to 10^5^ bacterial cells. Data providing the specificity of the designated primer sets is presented in Table 1. The qPCR results showed high specificity of the *plc* primer set for the detection of *C. perfringens* at the species level. There was no amplification with any of the DNA samples extracted from non-target microorganisms, thereby authenticating the specificity of the primer pair designed *in silico*. In case of *cpe*-targeted primer, the assay yielded positive reaction only with three strains i.e. ATCC 12917, ATCC 14809 and C052-1 that are known to harbor the functional chromosomal *cpe* gene (Table 1). *C. perfringens* ATCC 27324 also carry *cpe* gene on plasmid, but this gene is non-functional due to some specific mutation [42]. Notably, the *cpe*-specific primer (GAP 11/12) did not react with this strain (Table 1), verifying that this primer targets the conserved chromosomal sequence and hence detects only the functional *cpe* gene.

**Table 1:** *C. perfringens* targeted primers used in this study

Target	Standard strain	Primer	Sequence (5' - 3')	Product size (bp)	Annealing temp (°C)	Reference
*plc* gene	*C. perfringens* ATCC 13124^T^	Cper-plc508-F	CCGTTGATAGCGCAGGACA			
		Cper-plc508-R	CCCAACTATGACTCATGCTAGCA	219	60	This study
*cpe* gene	*C. perfringens*	GAP11	GGTTCATTAATTGAAACTGGTG	154	55	[37]
		GAP12	AACGCCAATCATATAAATTACAGC			
16S rRNA gene	*C. perfringens* ATCC 13124^T^	s-Clper-F	GGGGGTTTCAACACCTCC	170	60	[38, 74]
		ClPER-R	GCAAGGGATGTCAAGTGT			

### Determination of Amp-qPCR sensitivity

**Fig. 1:**
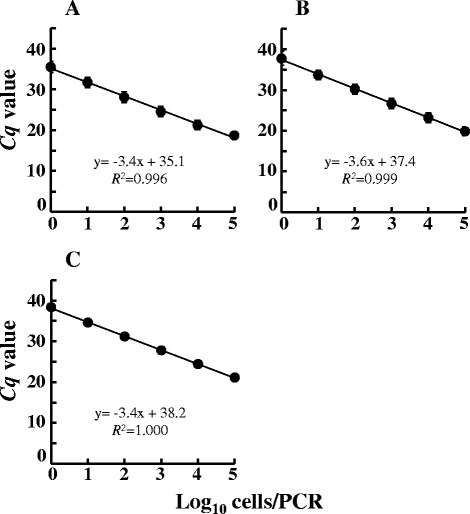
Standard curves representing the quantitative detection of reference strains of *C. perfringens* by Amp-qPCR assay. *C. perfringens* ATCC 13124^T^, ATCC 9856, ATCC 3624, ATCC 3626, ATCC 12917, ATCC 14809, ATCC 27324, and CS 052–1 were cultivated separately in Glu-mGAM. DNA fractions were extracted from the culture samples in the early stationary phase (24 h), and bacterial counts were determined microscopically with DAPI staining. 10-fold serial dilutions of DNA corresponding to the bacterial counts ranging from 10^0^ to 10^5^ bacterial cells were assessed by 16S rRNA gene-specific **a**, *plc*-specific **b**, and *cpe*-specific **c** Amp-qPCR assays. The *Cq* values obtained were plotted against the log_10_ number of bacterial cells subjected to each reaction. Data are expressed as means and standard deviations of the results from 7 strains (ATCC 13124^T^, ATCC 9856, ATCC 3624, ATCC 3626, ATCC 12917, ATCC 14809, and ATCC 27324) in the 16S rRNA gene-specific and *plc*-specific primer sets, and 3 strains (ATCC 12917, ATCC 14809, and CS 052–1) in the *cpe*-specific primer set

The sensitivity of three Amp-qPCR (qPCR using Ampdirect® Plus buffer) assays was tested with serial dilutions of DNA samples extracted from respective pure cultures of *C. perfringens* strains. When the *C*_*q*_ values were plotted against the log_10_ values of the initial number of *C. perfringens* cells in the qPCR to generate a standard curve, a standard linearity of regression curves was observed (Fig. 1). The counts of different *C. perfringens* strains in the pure culture obtained by DAPI staining (*x* axis) showed good correlation with the corresponding *C*_*q*_ values obtained by the respective Amp-qPCR assay (*y* axis) (Fig. 1; *R*^*2*^ > 0.99). All raw data i.e., *C*_*q*_ of the NTCs and LOD, analytical curve and PCR efficiency are provided in Additional file 1: Table S1 in Supplementary Material.

### Quantitative detection of *C. perfringens* spiked in fecal samples

**Fig. 2:**
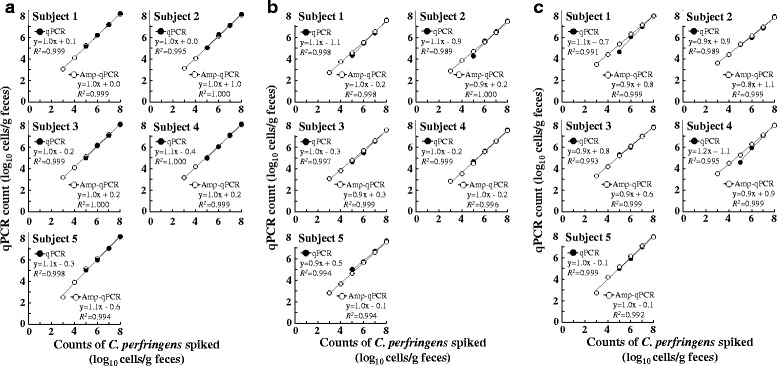
Quantitative detection of *C. perfringens* spiked in human feces by **a** 16S rRNA gene-specific, **b** *plc*-specific, and **c** *cpe*-specific qPCR assays with or without Ampdirect® Plus buffer. Strain ATCC 13124^T^ was used as reference for 16S rRNA gene- and *plc*-specific assays, and ATCC 12917 was used for *cpe*-specific assay

Cell counts from the 24 h old cultures were determined by DAPI staining using fluorescent microscopy, and serial dilutions of respective cultures were spiked separately into the fecal samples from five healthy volunteers who had been confirmed in advance by previously established 16S rRNA gene-specific assay to be Amp-qPCR-negative for *C. perfringens* (< 10^3^ bacterial cells/g feces) in their feces [38]. The *C. perfringens* counts obtained by qPCR as well as Amp-qPCR assays were well correlated with the expected values (*R*^*2*^ > 0.98; Fig. 2). The qPCR and Amp-qPCR yielded comparable slopes of the fitted curves, indicating that the two reactions gave similar amplification efficiencies. However, while the LOD of all the three qPCR assays (without Ampdirect® Plus) was about 10^5^ bacterial cells/g feces, it was lowered to 10^3^ bacterial cells/g with the use of Ampdirect® Plus PCR buffer (Amp-qPCR), indicating that the Amp-qPCR assays were about 100 times more sensitive than the qPCR assays for the quantitative detection of *C. perfringens* in fecal samples. Apparently, due to its ability to neutralize the effect of PCR inhibitors, the inclusion of Ampdirect® Plus buffer led to the use of more amount of stool DNA extract (maximum template amount of 1/20 in Amp-qPCR vs. 1/2,000 in qPCR) and hence a higher detection sensitivity. The counts of spiked *C. perfringens* (*x* axis) and the corresponding counts determined by Amp-qPCR assay (*y* axis) correlated well over the bacterial concentrations ranging from 10^3^ to 10^8^ bacterial cells/g of feces (*R*^*2*^ > 0.99; Fig. 2). These results indicated the efficiency and validity of these Amp-qPCR assays for the specific quantification of 16S rRNA, *plc* and *cpe* genes of *C. perfringens* in human feces.

### Detection of *C. perfringens* in stool samples

The Amp-qPCR assays were finally performed to enumerate the populations of *C. perfringens* in fecal samples from apparently healthy infants (*n* 124) and adult subjects (*n* 221). Table 3 shows the average counts and detection rates (%) of *C. perfringens* in the two cohorts, as determined by the Amp-qPCR assays. With the use of newly developed *plc*-specific Amp-qPCR assay, *C. perfringens* was detected (>10^3^ bacterial cells/g feces) in 36 % (44/124) of the infants and 33 % (70/221) of the adults, and its count ranged widely (10^3^ to 10^7^ bacterial cells/g feces) among each of the cohorts. The average *plc*^+^*C. perfringens* count was significantly (*p* = 0.00008) higher in infants (6.0 ± 1.5 log_10_ bacterial cells/g), as compared to that in adults (4.8 ± 1.2 log_10_ bacterial cells/g). The mean counts obtained by *plc*-specific Amp-qPCR assay were comparable with those obtained by 16S rRNA-targeted Amp-qPCR assay (Table 2). Accordingly, matching with the results of *plc*-specific assay, the average *C. perfringens* count analyzed by 16S rRNA-targeted assay was also significantly higher (*p* = 0.000001) in the infant cohort (6.4 ± 1.3 log_10_ bacterial cells/g), as compared to that in the adult cohort (4.8 ± 1.2 log_10_ bacterial cells/g). Although, at individual level, slight numerical differences between the counts analyzed by the two assays were observed in few samples (Additional file 2: Table S2) which can explain the minor difference in the detection rates analyzed by the two assays (Table 2), these differences were always less than one log value and could be attributed to the minor differences in the specificity and sensitivity of two different primers towards two different genes in different PCR reactions.

**Table 2:** Results of specificity tests using *plc*- and *cpe*-targeted primer sets

Taxon	Strain	Reactions with the following primer sets	
		Cper-plc508-F/R	GAP11/12
		(*plc*-target)	(*cpe*-target)
*Clostridium perfringens*	ATCC 13124^T^	+	–
*Clostridium perfringens*	ATCC 9856	+	–
*Clostridium perfringens*	ATCC 3624	+	–
*Clostridium perfringens*	ATCC 3626	+	–
*Clostridium perfringens*	ATCC 12917	+	+
*Clostridium perfringens*	ATCC 14809	+	+
*Clostridium perfringens*	ATCC 27324	+	–
*Clostridium perfringens*	C052-1	+	+
*Clostridium ramosum*	JCM 1298^T^	–	–
*Clostridium innocuum*	DSM 1286^T^	–	–
*Clostridium cocleatum*	JCM 1397^T^	–	–
*Clostridium symbiosum*	JCM 1297^T^	–	–
*Clostridium ghonii*	JCM 1400^T^	–	–
*Clostridium aminovalericum*	JCM 11016^T^	–	–
*Clostridium indolis*	JCM 1380^T^	–	–
*Clostridium paraputrificum*	JCM 1293^T^	–	–
*Clostridium butylicum*	JCM 1391^T^	–	–
*Clostridium difficile*	DSM 1296^T^	–	–
*Clostridium sordellii*	JCM 3814^T^	–	–
*Clostridium glycolicum*	JCM 1401^T^	–	–
*Clostridium lituseburense*	JCM 1404^T^	–	–
*Clostridium bifermentans*	JCM 1386^T^	–	–
*Blautia producta*	JCM 1471^T^	–	–
*Faecalibacterium prausnitzii*	ATCC 27768^T^	–	–
*Bacteroides vulgatus*	ATCC 8482^T^	–	–
*Collinsella aerofaciens*	DSM 3979^T^	–	–
*Prevotella melaninogenica*	ATCC 25845^T^	–	–
*Bifidobacterium longum*	ATCC 15707^T^	–	–
*Clostridium innocuum*	DSM 1286^T^	–	–
*Fusobacterium russii*	ATCC 25533^T^	–	–

### Detection of enterotoxin (*cpe*) gene-positive *C. perfringens*

The samples from the two cohorts were also analyzed by *cpe*-specific Amp-qPCR assay for the quantification of enterotoxigenic *C. perfringens* (Table 2). The carriage rate of *cpe*-positive *C. perfringens* was 10 % (12/124) in the infants which was significantly (*p* = 0.0004) higher as compared to that in the adults (1 %; 3/221). The average fecal count of *cpe*-positive *C. perfringens* in these positive samples was insignificantly (*p* = 0.536) higher in the infants (5.9 ± 1.9 log_10_ bacterial cells/g feces), as compared to that in the adults (4.8 ± 0.8 log_10_ bacterial cells/g feces). As expected, all the *cpe*-positive samples were also found to be positive by *plc*- and 16S rRNA gene-specific assays, and in most cases, the counts analyzed by *cpe*-specific assay were comparable to the counts analyzed by *plc*- and 16S rRNA gene-specific assays. In few cases, however, the count of *cpe*-positive *C. perfringens* was found to be lower than the counts analyzed by *plc*- or 16S rRNA-specific assays (Additional file 2: Table S2), which may be due to the presence of more than one strains of *C. perfringens* in these subjects.

**Table 3:** Quantitative detection of *C. perfringens* in stool samples from healthy infants and young adults by Amp-qPCR assays targeting 16S rRNA, *plc* and *cpe* genes

Target	Infant aged 6 months		Young adult	
	(*n* = 124)		(*n* = 221)	
	Bacterial count (log_10_ bacterial cells/g feces)^a^	Detection rate (%)	Bacterial count (log_10_ bacterial cells/g feces)^a^	Detection rate (%)
16S rRNA gene	6.4 ± 1.3	35	4.8 ± 1.2^b^	31
*plc*	6.0 ± 1.5	36	4.8 ± 1.2^b^	33
*cpe*	5.9 ± 1.9	10	4.8 ± 0.8	1^c^

^a^Data are expressed as the means and standard deviations

^b^ *p* < 0.05 (vs. Infant aged 6 months), Mann–Whitney *U* test

^c^ *p* < 0.05 (vs. Infant aged 6 months), Fisher’s exact probability test

## Discussion

In order to serve as an analytical tool to investigate the intestinal carriage and prevalence of α-toxigenic and enterotoxigenic *C. perfringens*, we developed a quantitative real-time PCR assay for rapid (within 4–5 h) and sensitive detection of this subdominant but important gut inhabitant in human feces. The assay had high analytical specificity as well as sensitivity and should become an efficient, rapid and sensitive alternate method to conventional methods for the detection and quantification of *C. perfringens* directly from pure cultures, feces and other complex samples. Data on linear range as well as amplification efficiency is important in order to authenticate the precision of qPCR for quantitative enumerations. With a quantitative detection limit of 10^3^ bacterial cells/g feces, a linear range of six to seven orders of magnitude and an acceptable reaction efficiency, our Amp-qPCR assays demonstrated an accurate quantification of *C. perfringens* from DNA extracted from pure culture or spiked fecal homogenates (Figs. 1, 2; Additional file 1: Table S1). We have already reported in our previous study of *C. difficile* that our in-house validated nucleic acid extraction method could extract RNA from the whole clostridial cells, i.e., in both the states, vegetative cells as well as spores [43]. Although we used DNA material in the present study, we followed same steps for the mechanical disruption of cells and for purification and precipitation of the nucleic acid material. Thus, our assay could detect whole bacterial cells of *C. perfringens* in feces and should be suitable for bacterial quantification irrespective of the bacterial growth phase. Overall, the assays developed in this study demonstrates threefold advantage: (a) high detection sensitivity of up to 10^3^ bacterial cells/g feces (which is significant for the detection of a subdominant gut microbe), (b) targeting chromosomal toxin gene (which is considerably important in context of a potential opportunistic pathogen), and (c) extraction of nucleic acids directly from feces (which makes the assay comparatively rapid). These assays were also used to quantify *C. perfringens* in samples from 124 infants and 221 adult subjects. The counts of *C. perfringens* in many subjects were found to be in a low range (i.e., 10^3^-10^4^ bacterial cells/g feces), signifying the importance of lower detection limit of 10^3^ bacterial cells/g feces of our assays for the investigation of fecal carriage of toxigenic *C. perfringens*, particularly in asymptomatic subjects. To our knowledge, this is the first report (a) to validate a culture-independent qPCR assay to quantify α-toxigenic and enterotoxigenic *C. perfringens* in human feces with an analytical sensitivity of 10^3^ bacterial cells/g feces, and (b) to report the intestinal carriage of these toxigenic *C. perfringens* strains in healthy Japanese infants and adults.

The fecal carriage of *C. perfringens* has earlier been studied (mainly in context to food-borne outbreaks), but the information about the toxigenic strains in asymptomatic subjects is limited [44–47]. It has generally been observed that healthy people carry less than 10^5^*C. perfringens* cfu/g feces while patients may carry 10^6^ or more cfu/g [44–47]. According to our data, *C. perfringens* may be present as commensal in the order of 10^6^-10^8^ bacterial cells/g feces even among healthy subjects. We detected a *plc*^+^*C. perfringens* count of more than 10^6^ bacterial cells/g in 21.7 % infants and 7.2 % adult subjects. This finding of higher carriage of *C. perfringens,* as compared to the previous studies, may be attributed to different study populations and/ or to the differences in detection sensitivities of different methods used. We targeted the α-toxin gene to make the assay specific for all the toxigenic *C. perfringens* strains (particularly in human feces), since α-toxin is produced by almost all the strains of *C. perfringens* and has been implicated in numerous gastrointestinal illnesses [4, 17, 48]. The counts analyzed by *plc*- and 16S rRNA gene-specific assays were comparable (Table 2), indicating that all the *C. perfringens* strains detected in this study harbored the α-toxin gene. This finding is consistent with those of prior studies [11, 17, 18, 30, 49]. Enterotoxigenic *C. perfringens* is responsible for nearly 70 % of *C. perfringens* food-poisoning outbreaks and 20 % of all non-food-borne gastrointestinal diseases [17, 50–53]. However, its ecology still remains under-explored, because of the widespread environmental distribution of *C. perfringens*, a very low ratio of *cpe*^+^ strains, and inability to quantify low count by majority of conventional methods (< 10^5^ bacterial cells/g) of toxigenic strains [5]. In this context, our sensitive culture-independent assays could be particularly important in clinical diagnosis or epidemiological investigation. Since *cpe*^+^ strains are also implicated in antibiotic-associated diarrhea, our assay should also find application in the analysis of such cases.

In our study, the average *plc*^+^*C. perfringens* count in infant cohort was found to be significantly higher than that in adult subjects (Table 2). Furthermore, the prevalence of *cpe*^+^*C. perfringens* was also significantly higher than that in adult subjects (Table 2). Generally, about 1–5 % of *C. perfringens* isolates produce enterotoxin [17, 52, 54, 55], however, in our study, the proportion of *cpe*-positive subjects out of the *C. perfringens-*positive ones was 27 % in infants, which was significantly (*p* = 0.001) higher as compared to that in adult subjects (4 %). This finding of higher fecal carriage of *cpe*^+^*C. perfringens*, particularly in infants, as compared to previous studies, may again be due to the differences in detection sensitivities of different methods used and/ or different study populations, since previous studies were mainly based on *C. perfringens* pure cultures or isolates from foods products and adult subjects. Nonetheless, the information on the occurrence of high population levels of toxigenic *C. perfringens* in infants’ gut is particularly relevant given the importance of such opportunistic pathogens in hosts with immature or compromised immune system [56–58]. It is well known that *C. perfringens* can colonize the infant gut in early postnatal period [59, 60], however it remains to be identified if the source of toxigenic *C. perfringens* strains in infants is from mother, hospital, foods or from surrounding environment, people, pets etc. Although *C. perfringens* enterotoxin occasionally causes diarrhea, most infants remain healthy and hence may be considered as asymptomatic carrier of toxic pathogen [52]. Nevertheless, further investigations are needed to elucidate why and how the prevalence of this pathogen diminishes as these infants grow up. It might be correlated to the maturation of the gastrointestinal microbial ecosystem and the associated immune system from infancy to adulthood [60–65], given that *C. perfringens* has been found to be more abundant in neonates and elderly than in adults [28, 66, 67].

We noticed that the counts of *cpe*^+^*C. perfringens* varied widely from 10^3^ to 10^8^ cells/g feces; and in most cases, the counts analyzed by *cpe*-, *plc*- or 16S rRNA-specific assays were comparable. Given the usefulness of fecal counts of *cpe*^+^*C. perfringens*, particularly the counts of > 10^5^ or > 10^6^ cfu/g, in the etiology of associated diseases [68], our findings of higher counts of *cpe*^+^*C. perfringens* in some healthy subjects may be an interesting target for future studies. Further, the differences in the counts analyzed by *plc*- and *cpe*-specific assays in some samples indicate that the intestinal carriage of *C. perfringens* may be diverse and that some healthy subjects may also act as potential reservoir of more than one *C. perfringens* strains. Such existence of *cpe*^+^*C. perfringens* has earlier been reported [17, 52, 53]; however, the significance of such human reservoirs in context to the disease/ risk transmission remains to be comprehended. While it remains uncertain whether toxigenic *C. perfringens* strains are primary pathogens or secondary intruders of injured intestinal mucosae, from our data of healthy subjects, it appears implausible that these strains are primary gastrointestinal pathogens. However, it cannot be ruled out that some healthy subjects may serve as a reservoir for *plc*^+^ and *cpe*^+^*C. perfringens* and hence may be more prone to developing *C. perfringens*-associated diseases, particularly in case of impaired intestinal mucosa and immunity or dysbiosis.

Several assays have earlier been developed for the detection of *C. perfringens*; however, most of these were mainly targeted for contaminated food products [13, 69, 70]. For example, the real-time PCR method developed by Fukushima et al. [69] could detect 10^1^ to 10^2^ cfu/g of food-borne pathogens in contaminated samples, but the PCR assay was preceded by a combination of filtration and low- and high-speed centrifugation protocols for the separation and concentration of bacteria from contaminated food samples such as chicken, pork, beef etc. and may not be competently applicable to human feces due to complex background microbiota, inhibitory substances and different matrixes. The assay developed by Kaneko et al. [13] could detect >10^3^ cfu/g of *cpe*-positive *C. perfringens* in meat samples, but it required enrichment culture specimens of spiked samples. dela Cruz et al. [29] developed a rapid real-time fluorescence resonance energy transfer PCR targeting *plc* and *cpe* genes of *C. perfringens*. However, the assay could detect about 20 copies of target sequence per PCR, whereas our assay could detect even a single copy per PCR using pure culture. Few studies have also targeted bacterial detection from feces of animals such as pigs and poultry, but the detection limit of these assays was about 10^4^ cfu/g feces [71, 72]. To detect *C. perfringens* in cattle feces, Gurjar et al. [73] developed a multiplex PCR assay with a detection limit of 30 pg and 5 pg of DNA for *plc* and *cpe* genes, respectively; however, the assay required pre-enrichment of the fecal samples to detect such low levels. Rinttila et al. [31] developed an array of real-time PCR methods for the analysis of 12 enteric pathogens including alpha-toxigenic *C. perfringens* from the human feces; however, the detection limit was set to 10^4^ bacterial genomic equivalents per gram of sample. In these contexts, our assay should be particularly advantageous for large-scale investigations of epidemiology and gut microbial ecology involving human samples since it can detect as low as 10^3^ toxigenic *C. perfringens* bacterial cells per gram of human feces without requiring any culturing step.

In summary, we have provided a novel qPCR assay for sensitive quantification of *C. perfringens* in human feces. We believe that the assay will represent a valuable analytical tool to quantify this subdominant but significant member of gut microbiota in human samples and will help in enhancing our understanding of its prevalence or pathogenesis. In addition, the assay should also be utilized in circumstances beyond fecal samples, and expedited detection should also be applicable in assessing foods, water, blood and other biological/ environmental samples. It is a limitation of our study that we did not examine *C. perfringens*-specific toxins, antitoxins or their bioactivities, and hence further studies on healthy and diseased subjects should be able to elucidate the clinical significance of high carriage of toxigenic *C. perfringens* strains in context to gastrointestinal health and microbiota.

## Additional files

Additional file 1: Table S1. Standard curve information. (PDF 13 kb) Additional file 2: Table S2. Quantitative data of *C. perfringens* count in stool samples from healthy infants and young adults by Amp-qPCR assays targeting 16S rRNA, *plc* and *cpe* genes. (PDF 52 kb)

## Abbreviations

Amp: Ampdirect® Plus PCR buffer
*Cq*: Quantification cycle
*Cpe*: *C. perfringens* enterotoxin
DAPI: 4’,6-diamidino-2-phenylindole
JCM: Japan Collection of Microorganisms
LOD: Limit of detection
MIQE: Minimum Information for the publication of real-time Quantitative PCR Experiments
NTC: No-template control
PCR: Polymerase chain reaction
qPCR: Quantitative PCR
RT-qPCR: Reverse transcriptase quantitative PCR
*plc*: Phospholipase C
