# Supplementary material for: Sensitive quantification of Clostridium perfringens in human feces by quantitative real-time PCR targeting alpha-toxin and enterotoxin genes
Source: BMC Microbiol. 2015 Oct 19;15:219. doi: 10.1186/s12866-015-0561-y (PMC4615878; doi:10.1186/s12866-015-0561-y)
Supplement: Additional file 1: Table S1. — Standard curve information. (PDF 13 kb) [file 12866_2015_561_MOESM1_ESM.pdf]

Supplementary Table S1 Standard curve information

Target	Primer name	Ampdirect® plus	Analytical curve	Correlation coefficient	Detection limit (log_{10} cells/reaction)	C*q* value for LOD	C*q* value for NTC	PCR efficiency
plc	Cper-plc508-F Cper-plc508-R	+	y= -3.4x + 36.8	0.9999	0	36.7	>50	0.95
		-	y= -3.3x + 31.7	0.9981	0	31.7	40.0	1.03
cpe	GAP11 GAP12	+	y= -3.4x + 38.5	0.9993	0	38.7	>50	0.98
		-	y= -3.5x + 34.8	0.9995	0	34.7	>50	0.94
16S rRNA gene	s-Clper-F ClPER-R	+	y= -3.4x + 34.9	0.9962	0	35.3	>50	0.97
		-	y= -3.2x + 30.3	0.9940	0	30.6	45.2	1.06
