# Supplementary material for: Sensitive quantification of Clostridium perfringens in human feces by quantitative real-time PCR targeting alpha-toxin and enterotoxin genes
Source: BMC Microbiol. 2015 Oct 19;15:219. doi: 10.1186/s12866-015-0561-y (PMC4615878; doi:10.1186/s12866-015-0561-y)
Supplement: Additional file 2: Table S2. — Quantitative data of C. perfringens count in stool samples from healthy infants and young adults by Amp-qPCR assays targeting 16S rRNA, plc and cpe genes. (PDF 52 kb) [file 12866_2015_561_MOESM2_ESM.pdf]

Supplementary Table S2 Quantitative data of *C. perfringens* count in stool samples from healthy infants and young adults by Amp-qPCR assays targeting 16S rRNA, *plc* and *cpe* genes

**Infant data (n=124)**					**Adult data (n=221)**				
		Bacterial count (Log10 bacterial cells/g feces)					Bacterial count (Log10 bacterial cells/g feces)		
		Amp-qPCR count					Amp-qPCR count		
S. No.	Sampl e ID.	*C. perfringens* 16S rRNA gene	*C. perfringens* *plc* gene	*C. perfringens* *cpe* gene	S. No.	Sample ID.	*C. perfringens* 16S rRNA gene	*C. perfringens* *plc* gene	*C. perfringens* *cpe* gene
1	1047	<3.1	<3.1	<3.1	1	7-3 (1)	<3.1	<3.1	<3.1
2	1097	<3.1	<3.1	<3.1	2	8-3 (2)	<3.1	<3.1	<3.1
3	1100	7.2	6.9	<3.1	3	9-3 (3)	4.7	4.8	<3.1
4	1107	<3.1	<3.1	<3.1	4	10-3 (4)	<3.1	<3.1	<3.1
5	1108	<3.1	<3.1	<3.1	5	11-3 (5)	7.7	7.8	<3.1
6	1109	4.4	4.3	<3.1	6	12-3 (6)	<3.1	<3.1	<3.1
7	1112	<3.1	3.2	<3.1	7	16-3 (7)	<3.1	<3.1	<3.1
8	1115	6.4	6.1	3.8	8	17-3 (8)	7.7	8.1	<3.1
9	1119	4.3	3.9	4.2	9	18-3 (9)	<3.1	<3.1	<3.1
10	1121	<3.1	<3.1	<3.1	10	20-3 (10)	<3.1	<3.1	<3.1
11	1122	7.6	7.3	<3.1	11	22-3 (11)	<3.1	<3.1	<3.1
12	1124	<3.1	<3.1	<3.1	12	1-3	<3.1	<3.1	<3.1
13	1129	<3.1	<3.1	<3.1	13	2-3	<3.1	<3.1	<3.1
14	1131	<3.1	<3.1	<3.1	14	3-3	<3.1	<3.1	<3.1
15	1133	7.2	6.9	<3.1	15	4-3	<3.1	<3.1	<3.1
16	1149	<3.1	<3.1	<3.1	16	5-3	<3.1	<3.1	<3.1
17	1150	<3.1	<3.1	<3.1	17	6-3	<3.1	<3.1	<3.1
18	1154	<3.1	<3.1	<3.1	18	7-3	<3.1	<3.1	<3.1
19	1155	<3.1	<3.1	<3.1	19	8-3	<3.1	<3.1	<3.1
20	1164	3.6	3.5	<3.1	20	9-3	<3.1	<3.1	<3.1
21	1172	7.7	7.4	8.4	21	10-3	3.1	3.6	<3.1

22	1174	<3.1	<3.1	<3.1	22	1211025	<3.1	<3.1	<3.1
23	1179	<3.1	<3.1	<3.1	23	1211026	<3.1	<3.1	<3.1
24	1180	<3.1	<3.1	<3.1	24	1211027	<3.1	<3.1	<3.1
25	1181	7.1	6.8	<3.1	25	1211028	3.8	3.4	<3.1
26	1183	<3.1	<3.1	<3.1	26	1211029	4.2	<3.1	<3.1
27	1184	<3.1	<3.1	<3.1	27	1211031	3.9	3.7	<3.1
28	1186	4.9	4.2	<3.1	28	1211032	<3.1	<3.1	<3.1
29	1188	<3.1	<3.1	<3.1	29	1211034	<3.1	<3.1	<3.1
30	1193	<3.1	<3.1	<3.1	30	1211035	<3.1	3.2	<3.1
31	1196	<3.1	<3.1	<3.1	31	1211036	3.3	3.8	<3.1
32	1204	5	4.7	5	32	1211038	<3.1	<3.1	<3.1
33	1208	7.3	7.0	<3.1	33	1211098	<3.1	<3.1	<3.1
34	1209	6.7	6.4	6.8	34	1211040	<3.1	<3.1	<3.1
35	1215	7.7	7.5	<3.1	35	1211041	5.0	5.4	<3.1
36	1216	<3.1	<3.1	<3.1	36	1211042	5.2	3.5	<3.1
37	1218	7.1	7.1	7.8	37	1211043	<3.1	3.5	<3.1
38	1220	7.2	7.0	<3.1	38	1211044	4.3	4.3	<3.1
39	1223	<3.1	<3.1	<3.1	39	1211046	<3.1	<3.1	<3.1
40	1226	<3.1	<3.1	<3.1	40	1211047	<3.1	<3.1	<3.1
41	1227	<3.1	<3.1	<3.1	41	1211048	<3.1	<3.1	<3.1
42	1228	<3.1	<3.1	<3.1	42	1211050	<3.1	<3.1	<3.1
43	1230	<3.1	<3.1	<3.1	43	1211051	<3.1	<3.1	<3.1
44	1231	<3.1	<3.1	<3.1	44	1211053	<3.1	<3.1	<3.1
45	1248	7	6.4	7.2	45	1211054	<3.1	<3.1	<3.1
46	1250	7.5	7.1	<3.1	46	1211055	<3.1	<3.1	<3.1
47	1257	<3.1	<3.1	<3.1	47	1211057	3.3	4	<3.1
48	1261	<3.1	<3.1	<3.1	48	1211058	<3.1	<3.1	<3.1
49	1267	<3.1	<3.1	<3.1	49	1211059	<3.1	<3.1	<3.1
50	1276	4.9	4.2	4	50	1211060	<3.1	<3.1	<3.1
51	1278	<3.1	<3.1	<3.1	51	1211061	<3.1	<3.1	<3.1
52	1280	8.1	7.6	<3.1	52	1211062	<3.1	3.2	<3.1
53	1287	<3.1	<3.1	<3.1	53	1211064	<3.1	<3.1	<3.1

54	1290	7.3	6.7	<3.1	54	1211066	<3.1	<3.1	<3.1
55	1291	<3.1	<3.1	<3.1	55	1211067	3.7	4	<3.1
56	1292	<3.1	<3.1	<3.1	56	1211068	<3.1	<3.1	<3.1
57	1301	<3.1	<3.1	<3.1	57	1211069	<3.1	<3.1	<3.1
58	1302	4.6	4.1	<3.1	58	1211072	3.8	4.1	<3.1
59	1308	5.9	5.2	<3.1	59	1211073	4.4	5	<3.1
60	1311	<3.1	<3.1	<3.1	60	1211074	3.3	4	<3.1
61	1312	<3.1	<3.1	<3.1	61	1211075	<3.1	<3.1	<3.1
62	1314	<3.1	<3.1	<3.1	62	1211076	3.2	3.8	<3.1
63	1316	6.7	6.4	<3.1	63	1211077	3.3	3.5	<3.1
64	1317	<3.1	<3.1	<3.1	64	1211079	<3.1	<3.1	<3.1
65	1324	5.8	5.2	<3.1	65	1211080	<3.1	<3.1	<3.1
66	1325	3.7	3.3	4.8	66	1211082	<3.1	<3.1	<3.1
67	1326	<3.1	<3.1	<3.1	67	1211083	<3.1	<3.1	<3.1
68	1332	<3.1	<3.1	<3.1	68	1211085	7.1	6.6	<3.1
69	1334	5.8	5.6	<3.1	69	1211086	<3.1	<3.1	<3.1
70	1335	<3.1	<3.1	<3.1	70	1211087	<3.1	<3.1	<3.1
71	1336	<3.1	<3.1	<3.1	71	1211088	<3.1	<3.1	<3.1
72	1338	4.6	4.4	<3.1	72	1211089	<3.1	<3.1	<3.1
73	1339	6.5	6.2	<3.1	73	1211091	<3.1	<3.1	<3.1
74	1341	<3.1	<3.1	<3.1	74	1211092	<3.1	<3.1	<3.1
75	1342	<3.1	<3.1	<3.1	75	1211093	6.7	6.3	<3.1
76	1343	7.3	7.1	<3.1	76	1211094	<3.1	<3.1	<3.1
77	1345	<3.1	<3.1	<3.1	77	1211095	<3.1	<3.1	<3.1
78	1347	6.8	6.3	7.3	78	1211096	4.5	4.1	<3.1
79	1349	<3.1	<3.1	<3.1	79	2111001	<3.1	<3.1	<3.1
80	1350	<3.1	<3.1	<3.1	80	2111002	<3.1	<3.1	<3.1
81	1352	<3.1	<3.1	<3.1	81	2111003	<3.1	<3.1	<3.1
82	1356	<3.1	<3.1	<3.1	82	2111036	<3.1	<3.1	<3.1
83	1357	<3.1	<3.1	<3.1	83	2111038	<3.1	<3.1	<3.1
84	1360	<3.1	<3.1	<3.1	84	2111040	<3.1	<3.1	<3.1
85	1362	<3.1	<3.1	<3.1	85	2111041	5.0	4	<3.1

86	1363	<3.1	<3.1	<3.1	86	2111042	<3.1	<3.1	<3.1
87	1365	<3.1	<3.1	<3.1	87	2111043	3.2	4	<3.1
88	1366	<3.1	<3.1	<3.1	88	2111045	5.4	5.6	<3.1
89	1368	<3.1	<3.1	<3.1	89	2111049	<3.1	3.4	<3.1
90	1369	<3.1	<3.1	<3.1	90	2111050	<3.1	<3.1	<3.1
91	1370	<3.1	<3.1	<3.1	91	2111054	<3.1	<3.1	<3.1
92	1372	6.9	6.6	<3.1	92	2111056	<3.1	7.9	<3.1
93	1373	<3.1	<3.1	<3.1	93	2111057	<3.1	<3.1	<3.1
94	1374	<3.1	<3.1	<3.1	94	2111060	<3.1	<3.1	<3.1
95	1375	4.2	3.8	4.4	95	2111062	<3.1	<3.1	<3.1
96	1376	<3.1	<3.1	<3.1	96	2111063	<3.1	<3.1	<3.1
97	1379	<3.1	<3.1	<3.1	97	2111064	4.8	5	<3.1
98	1382	6.7	6.3	<3.1	98	2111066	<3.1	<3.1	<3.1
99	1383	<3.1	<3.1	<3.1	99	2111067	<3.1	<3.1	<3.1
100	1386	3.0	<3.1	<3.1	100	2111068	8.1	8.2	<3.1
101	1388	7.6	7.4	<3.1	101	2111069	<3.1	<3.1	<3.1
102	1391	<3.1	<3.1	<3.1	102	2111070	<3.1	<3.1	<3.1
103	1392	<3.1	<3.1	<3.1	103	2111072	<3.1	<3.1	<3.1
104	1393	4.9	4.3	<3.1	104	2111073	<3.1	<3.1	<3.1
105	1394	5.9	5.3	<3.1	105	2111076	6.7	7.1	<3.1
106	1396	<3.1	<3.1	<3.1	106	2111077	<3.1	3.4	<3.1
107	1397	5.4	4.8	<3.1	107	2111078	<3.1	<3.1	<3.1
108	1399	<3.1	<3.1	<3.1	108	2111079	<3.1	<3.1	<3.1
109	1402	8.7	8.2	<3.1	109	2111083	6.3	6.3	<3.1
110	1404	<3.1	<3.1	<3.1	110	2111084	6.0	6	<3.1
111	1406	<3.1	<3.1	<3.1	111	2111085	4.3	4.2	<3.1
112	1407	<3.1	<3.1	<3.1	112	2111086	<3.1	<3.1	<3.1
113	1408	<3.1	<3.1	<3.1	113	2111088	4.5	4.1	<3.1
114	1409	<3.1	<3.1	<3.1	114	2111089	4.1	4	<3.1
115	1410	<3.1	<3.1	<3.1	115	2111091	<3.1	<3.1	<3.1
116	1411	<3.1	<3.1	<3.1	116	2111093	<3.1	<3.1	<3.1
117	1412	<3.1	<3.1	<3.1	117	2111095	<3.1	<3.1	<3.1

118	1413	<3.1	<3.1	<3.1
119	1414	7.5	6.7	7.4
120	1415	7.3	6.7	<3.1
121	1417	<3.1	<3.1	<3.1
122	1418	<3.1	<3.1	<3.1
123	1419	7.9	7.4	<3.1
124	1420	7.5	7.1	<3.1
Average		6.3	6.0	5.9
Std. Dev.		1.4	1.5	1.9
Det. rate, %		35.5 (44/124)	35.5 (44/124)	9.7 (12/124)

118	2111096	<3.1	<3.1	<3.1
119	2111097	6.3	6.4	<3.1
120	2111098	6.4	6.9	5.5
121	2111101	<3.1	<3.1	<3.1
122	2111102	<3.1	<3.1	<3.1
123	2111103	4.4	4	<3.1
124	2111106	4.9	4.8	<3.1
125	2111107	<3.1	<3.1	<3.1
126	2111108	<3.1	<3.1	<3.1
127	2111109	<3.1	<3.1	<3.1
128	2111110	<3.1	4.3	<3.1
129	2111111	<3.1	<3.1	<3.1
130	1311001	3.5	4	<3.1
131	1311002	<3.1	<3.1	<3.1
132	1311003	5.6	6	<3.1
133	1311005	<3.1	<3.1	<3.1
134	1311006	<3.1	<3.1	<3.1
135	1311007	<3.1	<3.1	<3.1
136	1311011	<3.1	<3.1	<3.1
137	1311014	<3.1	<3.1	<3.1
138	1311016	<3.1	<3.1	<3.1
139	1311017	<3.1	<3.1	<3.1
140	1311018	<3.1	3.8	<3.1
141	1311019	<3.1	<3.1	<3.1
142	1311023	6.0	6.4	3.9
143	1311025	<3.1	<3.1	<3.1
144	1311026	<3.1	<3.1	<3.1
145	1311027	<3.1	<3.1	<3.1
146	1311028	<3.1	<3.1	<3.1
147	1311031	4.6	<3.1	<3.1
148	1311033	<3.1	<3.1	<3.1
149	1311035	<3.1	<3.1	<3.1

150	1311036	6.9	6.3	<3.1
151	1311037	<3.1	<3.1	<3.1
152	1311038	5.1	5	<3.1
153	1311039	<3.1	<3.1	<3.1
154	1311040	5.6	5.9	<3.1
155	1311044	<3.1	<3.1	<3.1
156	1311045	3.7	4	<3.1
157	1311046	<3.1	<3.1	<3.1
158	1311047	<3.1	<3.1	<3.1
159	1311048	3.4	3.2	<3.1
160	1311049	3.6	4	<3.1
161	1311051	5.4	6.3	<3.1
162	1311053	5.2	4.9	<3.1
163	1311054	<3.1	<3.1	<3.1
164	1311056	<3.1	<3.1	<3.1
165	1311058	<3.1	<3.1	<3.1
166	1311059	5.9	5.8	<3.1
167	1311060	3.2	5.2	<3.1
168	1311065	<3.1	<3.1	<3.1
169	1311066	<3.1	<3.1	<3.1
170	1311068	4.0	3.6	<3.1
171	1311069	<3.1	<3.1	<3.1
172	1311070	5.7	5.7	<3.1
173	1411001	<3.1	<3.1	<3.1
174	1411002	<3.1	<3.1	<3.1
175	1411003	<3.1	<3.1	<3.1
176	1411004	<3.1	<3.1	<3.1
177	1411009	<3.1	<3.1	<3.1
178	1411010	<3.1	<3.1	<3.1
179	1411011	<3.1	<3.1	<3.1
180	1411013	<3.1	<3.1	<3.1
181	1411014	<3.1	<3.1	<3.1

182	1411017	5.0	5.0	<3.1
183	1411018	<3.1	<3.1	<3.1
184	1411019	<3.1	<3.1	<3.1
185	1411021	<3.1	<3.1	<3.1
186	1411023	4.4	4.2	<3.1
187	1411024	<3.1	<3.1	<3.1
188	1411025	<3.1	<3.1	<3.1
189	1411026	4.3	4.7	<3.1
190	1411027	<3.1	<3.1	<3.1
191	1411028	5.2	5.2	<3.1
192	1411029	<3.1	<3.1	<3.1
193	1411030	5.5	5.5	<3.1
194	1411031	<3.1	<3.1	<3.1
195	1411033	<3.1	<3.1	<3.1
196	1411036	4.6	4.7	<3.1
197	1411037	<3.1	<3.1	<3.1
198	1411038	4.3	4.4	<3.1
199	1411041	<3.1	<3.1	<3.1
200	1411043	5.3	5.3	<3.1
201	1411046	<3.1	<3.1	<3.1
202	1411047	4.9	4.8	5.1
203	1411048	<3.1	<3.1	<3.1
204	1411049	<3.1	<3.1	<3.1
205	1411051	<3.1	<3.1	<3.1
206	1411052	<3.1	<3.1	<3.1
207	1411054	4.9	4.8	<3.1
208	1411055	4.1	4.1	<3.1
209	1411056	4.5	5	<3.1
210	1411057	<3.1	<3.1	<3.1
211	1411058	<3.1	<3.1	<3.1
212	1411059	5.8	5.7	<3.1
213	1411060	4.0	4.2	<3.1

214	1411061	5.4	5.5	<3.1
215	1411063	<3.1	<3.1	<3.1
216	1411064	<3.1	<3.1	<3.1
217	1411065	<3.1	<3.1	<3.1
218	1411066	<3.1	<3.1	<3.1
219	1411067	<3.1	<3.1	<3.1
220	1411068	6.0	6.2	<3.1
221	1411069	<3.1	<3.1	<3.1
Average		4.8	4.8	4.8
Std. Dev.		1.1	1.2	0.8
Det. rate, %		29.0<br>(64/221)	31.7<br>(70/221)	1.4<br>(3/221)
